# Identification of Novel Variants in *LTBP2* and *PXDN* Using Whole-Exome Sequencing in Developmental and Congenital Glaucoma

**DOI:** 10.1371/journal.pone.0159259

**Published:** 2016-07-13

**Authors:** Shazia Micheal, Sorath Noorani Siddiqui, Saemah Nuzhat Zafar, Aftab Iqbal, Muhammad Imran Khan, Anneke I. den Hollander

**Affiliations:** 1 Department of Ophthalmology, Radboud University Medical Centre, Nijmegen, the Netherlands; 2 Department of Pediatric Ophthalmology, Al-Shifa Eye Trust Hospital Jhelum Road, Rawalpindi, Pakistan; 3 School of Biosciences, University of Westminster, London, United Kingdom; 4 Department of Human Genetics, Radboud University Medical Centre, Nijmegen, the Netherlands; CNR, ITALY

## Abstract

**Background:**

Primary congenital glaucoma (PCG) is the most common form of glaucoma in children. PCG occurs due to the developmental defects in the trabecular meshwork and anterior chamber of the eye. The purpose of this study is to identify the causative genetic variants in three families with developmental and primary congenital glaucoma (PCG) with a recessive inheritance pattern.

**Methods:**

DNA samples were obtained from consanguineous families of Pakistani ancestry. The *CYP1B1* gene was sequenced in the affected probands by conventional Sanger DNA sequencing. Whole exome sequencing (WES) was performed in DNA samples of four individuals belonging to three different *CYP1B1*-negative families. Variants identified by WES were validated by Sanger sequencing.

**Results:**

WES identified potentially causative novel mutations in the latent transforming growth factor beta binding protein 2 (*LTBP2*) gene in two PCG families. In the first family a novel missense mutation (c.4934G>A; p.Arg1645Glu) co-segregates with the disease phenotype, and in the second family a novel frameshift mutation (c.4031_4032insA; p.Asp1345Glyfs*6) was identified. In a third family with developmental glaucoma a novel mutation (c.3496G>A; p.Gly1166Arg) was identified in the *PXDN* gene, which segregates with the disease.

**Conclusions:**

We identified three novel mutations in glaucoma families using WES; two in the *LTBP2* gene and one in the *PXDN* gene. The results will not only enhance our current understanding of the genetic basis of glaucoma, but may also contribute to a better understanding of the diverse phenotypic consequences caused by mutations in these genes.

## Introduction

Childhood or infantile glaucoma include primary congenital glaucoma (PCG) and developmental glaucoma, which can be associated with syndromes (e.g. Axenfeld Rieger syndromes) or can lead to defects only in the eye. The majority (about 60%) of patients with these types of glaucoma are diagnosed by the age of 6 months, and 80% are diagnosed within the first year of life. PCG usually leads to permanent visual impairment, and accounts for 0.01–0.04% of total blindness [1,2]. The incidence of PCG, however, varies among different populations: 1 in 2,500 in Saudi Arabians, 1 in 3,300 in southern Indians, 1 in 1,250 in Slovakian gypsies [3], and from 1 in 10,000 to 1 in 20,000 in Western populations [4]. In most cases, developmental anomalies affect the anterior chamber and the trabecular meshwork. PCG is characterized by an elevated intraocular pressure (IOP), an increased corneal diameter, and optic disc damage [4,5]. The clinical features of PCG include buphthalmos, corneal edema and opacification with rupture of the Descemet membrane, thinning of the anterior sclera, iris atrophy, and an anomalously deep anterior chamber. In addition, less common features include epiphora, blepharospasm, and photophobia.

Three chromosomal locations have been associated with PCG: GLC3A at 2p21, GLC3B at 1p36.2-1p36.1, GLC3C at 14q24.3, and thus far only two causative genes (*CYP1B1*, *LTBP2*) have been identified [6]. The *CYP1B1* gene is frequently mutated, ranging 20–100% in PCG cases from Japan, Saudi Arabia and Slovakian gypsies [7]. Mutations in the *PXDN* gene are known to cause developmental glaucoma with opacification/clouding of the cornea, cataract, and developmental glaucoma in Pakistani and Cambodian families [8].

In the current study, we aimed to identify novel mutations in three *CYP1B1*-negative glaucoma families from Pakistan using whole-exome sequencing (WES).

## Materials and Methods

### Subjects

The participants were recruited at the pediatric glaucoma department of Al-Shifa Eye Trust Hospital, Rawalpindi, Pakistan. Blood samples were collected from affected and unaffected siblings, and from the parents. Genomic DNA was extracted using AutoPure LS DNA Extractor and PUREGEN reagents (Gentra Systems Inc, Minneapolis, Minnesota, USA).

### Ethics statement

This study was approved by the Institutional Review Board of the Al-Shifa Eye Trust hospital, and adhered to the tenets of the Declaration of Helsinki. Written informed consent was obtained for study participation from the participants and/or their parents, as appropriate.

### WES and Sanger Sequencing

WES was employed to identify the disease-associated genes in four subjects, belonged to three families. Genomic DNA was prepared from peripheral venous blood, and was randomly fragmented into 200–400 base pair (bp) fragments with the Covaris Acoustic System according to the manufacturer’s instructions (Covaris, Inc., Woburn, MA). Kapa Library preparation (Kapa Biosystems, Inc., Wilmington, MA) was performed on a Caliper Sciclone NGS workstation (Caliper Life Sciences, Hopkinton, MA) followed by capture using the Nimblegen SeqCap EZ V2 kit (Roche Nimblegen, Inc., Madison, WI). Each captured library was then loaded on an Illumina HiSeq2000 sequencer using Illumina TruSeq V3 chemistry (Illumina, Inc., San Diego, CA).

Downstream analyses included demultiplexing (CASAVA software, Illumina), alignment to the human reference genome (GRCh37, UCSC hg19) using the Burrows-Wheeler alignment (BWA)[9] tool. Alignments were sorted by Picard (http://broadinstitute.github.io/picard) and subsequently processed by GATK [10].

Finally, the mean depth of coverage was determined using GATK, and Free mix values were estimated through verify BAMid [11]. Samples that passed technical QC metrics were genotyped to gVCF level through GATKs Haplotype Caller. Indels, SNVs, microinsertions and microdeletions were filtered separately using GATKs Variant-Quality Score Recalibration. Both the SNV and indel sets were annotated using ANNOVAR [12]. Only mapped reads were used for subsequent analysis. They were annotated with information from the University of California, Santa Cruz genome annotation database (http://genome.ucsc.edu/index.html), consensus coding sequence (http://www.ncbi.nlm.nih.gov/CCDS/CcdsBrowse.cgi), Ensembl (http://www.ensembl.org), RefSeq (http://www.ncbi.nlm.nih.gov/RefSeq/), MirBase (http://www.mirbase.org/), and EntrezGene (www.ncbi.nlm.nih.gov/entrez/query.fcgi?db=gene).

All variants obtained through WES were first filtered against several public databases for the minor allele frequency (MAF) < 0.5%, including dbSNP135, 1000 Genomes Project databases (http://www.1000genomes.org/, 1000g2012April_all version), NHLBI GO Exome Sequencing Project (ESP6500, http://evs.gs.washington.edu/EVS/, esp6500si_all version) and the Human Genetic Variation Database (HGVD,http://www.genome.med.kyoto-u.ac.jp/SnpDB/). Subsequently, only coding nonsynonymous variants, frameshift, and splice site variants were analyzed. To predict the functional impact of the sequence variants on the encoded protein, the pathogenicity of missense variants was evaluated by publically available tools including PhyloP, Grantham, polymorphism phenotyping v-2 (PolyPhen-2) (version 2.1.0 r367; http://genetics.bwh.harvard.edu/pph2/), MutationTaster, and sorting intolerant from tolerant (SIFT; http://sift.jcvi.org/).

Variant validation and segregations analysis of variants identified by WES were carried out by performing standard PCR and Sanger sequencing using ABI BigDye chemistry (Applied Biosystems Inc, Foster City, California, USA), and was processed through an automated ABI 3730 Sequencer (Applied Biosystems, Inc).

## Results

### Family 1

Proband IV:1 was a 5-year-old boy with buphthalmos in both eyes. He had a corneal diameter of 14.5 mm in the right eye and 13.5 mm in the left eye. The corneal thickness was 0.625 mm and 0.733 mm in the right and left eyes, respectively. Axial lengths were 26.32 mm in the right and 21.22mm in the left eye. Intraocular pressures (IOP) were 24 mmHg in the right and 25 mmHg in the left eye. In the left eye he had a persistant pupillary membrane which was removed by surgery. He had subluxated lenses in both eyes. The cup-disc ratio (CDR) was 0.7 in the right eye and 0.2 in the left eye.

Sibling IV:2 was a 3-year-old boy with a phthisical right eye after surgery for glaucoma, and a left eye with buphthalmos. The IOP of the left eye was 13 mmHg after surgical treatment and topical drops with a vision of 6/ 96, and the CDR was 0.9. He had microspherophakia (a small spherical lens) and an iridogoniodysgenesis (IGDS)-like iris pattern.

**Fig 1:**
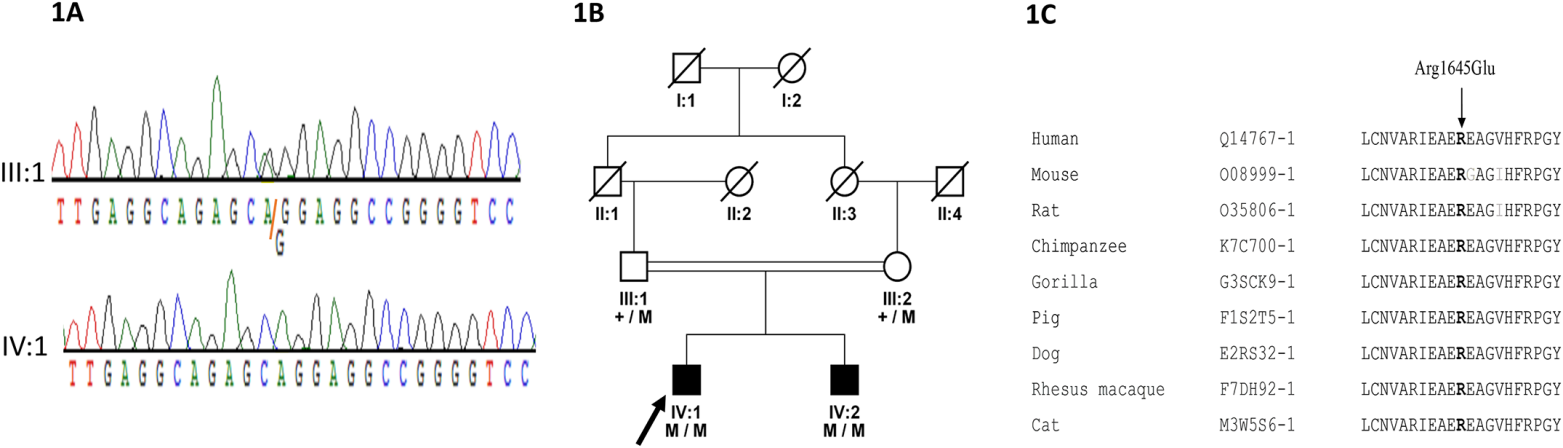
(a) Sanger sequencing chromatograms showing III:1 carrier and IV:1 homozygous mutant (b) Family pedigree and segregation of a novel missense mutation (c.4934G>A; p.Arg1645Glu) in the *LTBP2* gene in a PCG family. (c). Multiple sequence alignment of the region of the *LTBP2* protein surrounding the novel Arg1645Glu mutation in various species. The arginine residue (indicated with an arrow) is highly conserved among all species analyzed.

WES in DNA samples of the two affected children of the family revealed a novel pathogenic missense variant (c.4934G>A; p.Arg1645Glu) in *LTBP2* (Fig 1a and 1b). This particular variant was predicted to be deleterious by SIFT, damaging by Polyphen-2, and disease causing by MutationTaster. The PhyloP score was 3.64 and Grantham distance was 43. The mutated arginine amino acid was conserved among PRDM5 orthologs from different species (Fig 1c).

### Family 2

Proband IV:2 was a 4-year-old boy who visited the hospital with the complaint of watering eyes. On examination he had a hazy cornea, buphthalmos, and poor vision in both eyes. He had four surgeries for glaucoma. The IOP was 24 mmHg and 30 mmHg and the CDR was 0.7 and 0.9 for the right and the left eye, respectively.

**Fig 2:**
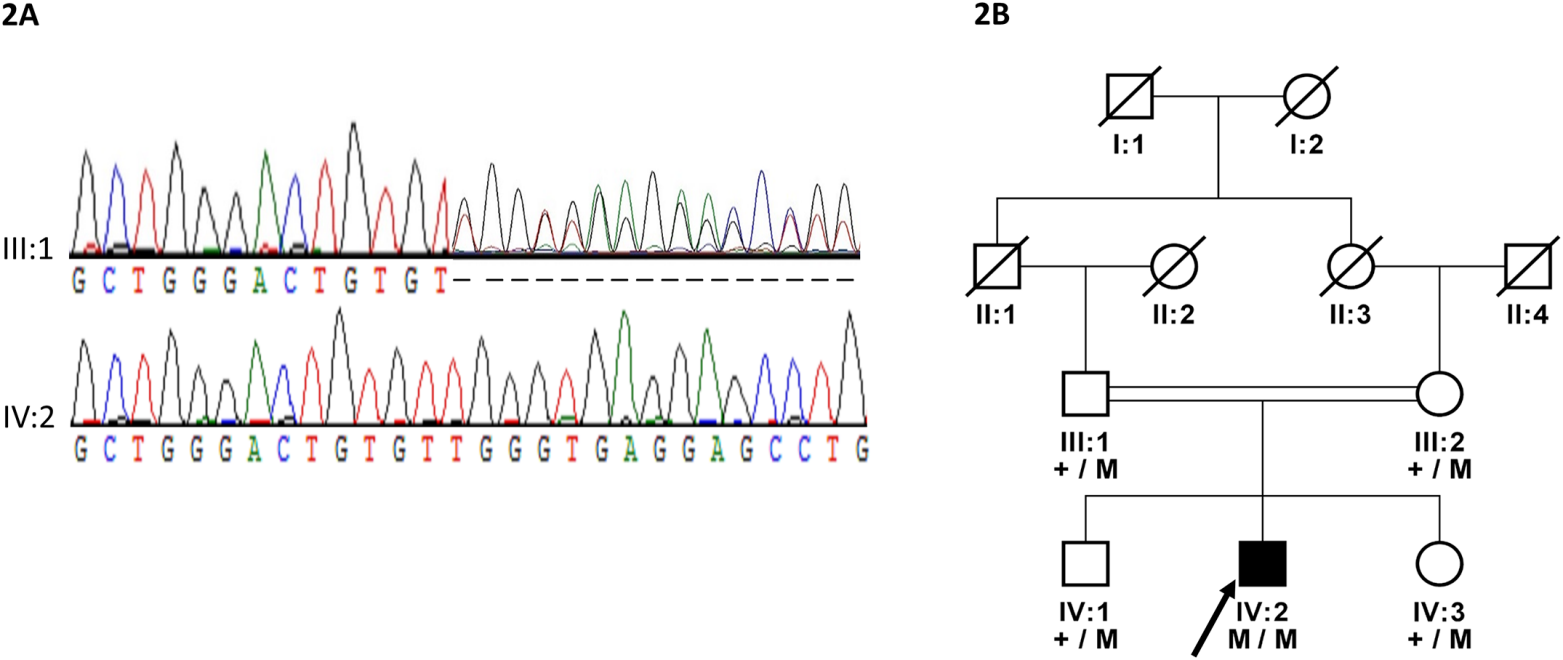
(a) Sanger sequencing chromatograms for the carrier III:1 and affected individual IV:2 homozygous for the mutation (b) Family pedigree and segregation of a novel frameshift mutation (c.4031_4032insA; p.Asp1345Glyfs*6) in the *LTBP2* gene in a PCG family.

In this family a novel homozygous frameshift mutation (c.4031_4032insA; p.Asp1345Glyfs*6) segregates with the disease (Fig 2a and 2b). Due to this single base pair insertion the reading frame is shifted leading to a premature stop codon, which may result in a truncated protein, or the the mRNA may be subjected to nonsense-mediated decay.

### Family 3

Proband IV:2 was a 9-year-old girl with a hazy cornea. Axial lengths were 19.7 mm and 17.3 mm with a horizontal corneal diameter of 11.5 mm and 10 mm for the right and the left eyes, respectively.

Sibling IV:3 was a 6-month old girl having a severe bilateral corneal haze, due to which detailed clinical evaluation of the anterior chamber was not possible. Her left eye was phthisical, and the right eye had poor vision due to corneal opacity. The IOP of the right eye was within normal range after surgery for glaucoma.

**Fig 3:**
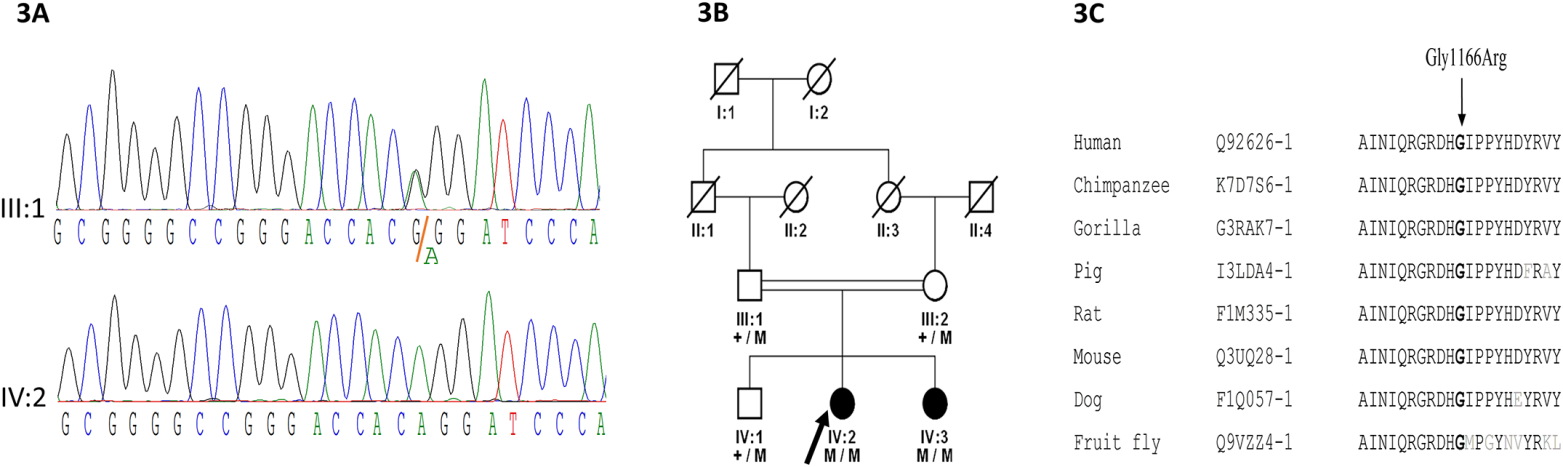
(a) DNA chromatogram of the relevant *PXDN* fragment for the carrier and homozygous variant are shown (b). Family pedigree and segregation of a novel missense mutation (c.3496G>A; p.Gly1166Arg) in the *PXDN* gene. (c). Multiple sequence alignment of the region of the *PXDN* protein surrounding the novel Gly1166Arg mutation in various species. The glycine residue (indicated with an arrow) is highly conserved among all species analyzed.

WES identified a novel missense variant (c.3496G>A; p.Gly1166Arg) in the *PXDN* gene (Fig 3a and 3b). This variant segregates with the disease phenotype in the family and is predicted to be deleterious, damaging and disease causing by SIFT, Polyphen-2, and MutationTaster, respectively, with a Grantham score of 125 and a PhyloP score of 6.10. The Gly1166Arg variant affects a glycine residue in the haem peroxidase domain of the protein, which is a highly conserved amino acid among *PXDN* orthologs of different species (Fig 3c).

The novel variants segregating with disease in *LTBP2* and *PXDN* have been excluded from 150 Pakistani healthy controls.

## Discussion

In the current study we identified two novel potentially pathogenic mutations (c.4934G>A; p.Arg1645Glu and c.4031_4032insA; p.Asp1345Glyfs*6) in the *LTBP2* gene in two PCG families from Pakistan. Exome data of the all patients can be viewed using the link: http://datadryad.org/review?doi=doi:10.5061/dryad.k7455. In the *PXDN* gene a novel mutation (c.3496G>A; p.Gly1166Arg) was identified in a family with developmental glaucoma. Previously, mutations in the *LTBP2* gene have been identified in the four recessive Pakistani families with PCG: a homozygous single base pair deletion in exon 1 (c.412 del G; p.Ala138Profs*278), a homozygous nonsense mutation in exon 4 (c.895C >T; p.Arg299X), a homozygous 14-base pair deletion in exon 6 (c.1243-1256 del; p.Glu415Argfs*596), and a homozygous nonsense variant in exon 1 (c.331C>T; p.Gln111X). In European Gypsies a recurrent founder mutation (c.895C >T; p.Arg299X) was identified in 8 patients, which was initially identified in a Pakistani PCG family, suggesting that they may have a common ancestry [13]. More recently, loss of function mutations (c.1415delC; p.Ser472Trpfs*3 and c.5376delC; p.Tyr1793Alafs*55) in the *LTBP2* gene have been identified in Iranian families [14]. In PCG patients from the United States, WES revealed 3 nonsynonymous and 11 synonymous sequence variants in the *LTBP2* gene in heterozygous state but since a second variant remained unidentified these variants are unlikely to be causative[15]. On the contrary, no variants in the *LTBP2* gene were identified in 54 Saudi Arabian families [16], in Turkish and British PCG families [17] nor in North Indian patients [18]. This suggests that *LTBP2* mutations are a relatively rare cause of PCG.

The *LTBP2* protein is expressed in the trabecular meshwork and ciliary processes involved in the regulation and production of the aqueous humor. In other tissues, *LTBP2* is involved in tissue repair and cell adhesion [19]. Mutations in *LTBP2* were identified in PCG families and patients from Pakistan and Gypsies [13]. The mechanism behind the pathogenic involvement of the *LTBP2* gene in glaucoma is not clearly established, but it has been demonstrated that the *LTBP2* protein is associated with elastic fibers in developing elastic tissues [20]. Furthermore, *LTBP2* interacts with fibrillin 1 (FBN1), which is required for its integration into the extracellular matrix [21]. The *LTBP2* protein binds directly to FBN1 and competes with LTBP1 for this interaction [22]. Mutations in the *FBN1* gene are known to cause Marfan syndrome, and a number of studies have linked homozygous mutations in the *LTBP2* gene with a syndrome including megalocornea, microspherophakia, lens dislocation, and secondary glaucoma developing after the age of 3 years [23] and in isolated microspherophakia/lens dislocation [24]. In addition to involvement in PCG, pathogenic mutations in the *LTBP2* gene have also been linked to Weill-Marchesani syndrome characterized by abnormalities of the lens of the eye, proportionate short stature, brachydactyly, and joint stiffness [25]. These reports, together with the current study, and the *LTBP2* expression pattern in the trabecular meshwork, ciliary body and ciliary process[13], shows underlying pathophysiology of *LTBP2* in different eye diseases.

Peroxidasin protein *(PXDN)* is localized to the cornea and in the layers of the lens epithelial. Previous studied identified that it is required for the normal development of the anterior chamber of the eye [8]. Therefore, any pathogenic change or mutations affect the normal development of the eye and results in various congenital eye anomalies. Previously, mutations in *PXDN* have been reported in families and patients with congenital cataracts, microcornea, sclerocornea, developmental glaucoma, and anterior segment dysgenesis [8,26]

In the current study, a novel missense mutation (c.3496G>A; p.Gly1166Arg) is identified in the *PXDN* gene in a Pakistani family with the developmental glaucoma. Mutations in the *PXDN* were previously reported to cause severe inherited eye diseases, such as congenital cataract, corneal opacity and developmental glaucoma in Pakistani [(c.2638C>T; p.Arg880Cys), (c.2568delC; p.Cys857Alafs*5)] and Cambodian (c.1021C>T; p.Arg341X) families [8]. In addition to homozgous mutations identified in the current and previous studies, recently, mutations in the *PXDN* have been identified compound heterozygously in a non-consanguineous family with two children who had anterior segment dysgenesis, sclerocornea, microphthalmia, hypotonia and developmental delays. Both siblings carried a heterozygous nonsense mutation (c.1021C>T; p.Arg341*) and a heterozygous 23-basepair deletion leading to a frameshift (c.2375_2397del23; p.Leu792Hisfs*67). In addition, a sporadic male patient carried a heterozygous frameshift mutation (c.1192delT; p.Tyr398Thrfs*40) and a heterozygous missense substitution (c.947 A>C; p.Gln316Pro) [26].

A *Pxdn* mutant mouse carrying a premature stop codon mutation was described to have a severe anterior segment dysgenesis and microphthalmia, resembling the phenotype in patients with *PXDN* mutations. In the mutant mice, proliferation and differentiation of the lens is disrupted due to the extrusion of lens material outside the lens, and aberrant expression of Pax6 and Foxe3 was observed. The defective peroxidasin was described to affect the structural integrity of the ocular basement membrane, causing damage to the anterior segment of the eye and leading to developmental eye defects. Moreover, the *PXDN* mutants exhibited an early-onset glaucoma and progressive retinal dysgenesis [27,28].

Both affected siblings in the current study had corneal opacity, glaucoma, and buphthalmos. The homozygous missense mutation (c.3496G>A; p.Gly1166Arg) affects a highly conserved glycine reside in the haem peroxidase domain of the protein. Therefore, it is likely that this variant affects the peroxidase activity of the protein, potentially leading to impaired integrity of the basement membrane.

The pathogenic mechanism involving both *LTBP2* and *PXDN* together in glaucoma is not clearly established, however it has been proposed that both of them are linked to each other via COL4A2 http://pathwaynet.princeton.edu/predictions/geneset/?network=human-functional-relation&geneset=4314%2C14540#. Collagens are present in the ECM together with other proteoglycans, glycoproteins (*LTBP2* and *PXDN*) fibronectin and fibrillin-1. The ECM assembly is important in the TM for regulating the IOP of the eye [28, 29]. The excessive accumulation of collagens might be able to disrupt their interaction with other proteins in the ECM or it could be the variants present in other proteins present in close contact with collagens, such as *LTBP2* and *PXDN*, alters the normal mechanism of ECM assembly and affect the anterior segment of the eye resulting in the vision impairment due to defects in the anterior parts of the eye such as iris, cornea, Descemet membrane etc.

In summary, we identified two novel mutations (c.4934G>A; p.Arg1645Glu and c.4031_4032insA; p.Asp1345Glyfs*6) in the *LTBP2* gene and one novel mutation (c.3496G>A; p.Gly1166Arg) in the *PXDN* gene in Pakistani families with the PCG and developmental glaucoma. Our study, together with previous published studies, suggests that both *LTBP2* and *PXDN* are essential for eye development, and are important members of the extracellular matrix essential for basement membrane integrity and cell adhesion during the eye development. Mutations in both *LTBP2* and *PXDN* can cause diverse phenotypic consequences, therefore, more studies are required to contribute to an accurate phenotypic classification based on the genetic diagnosis.

## References

[1] MD S (2005) Shields’ Textbook of Glaucoma. New York: Lippincott Williams and Wilkins publishers.

[2] Gould DB, John SW (2002) Anterior segment dysgenesis and the developmental glaucomas are complex traits. Hum Mol Genet 11: 1185–1193. 12015278 10.1093/hmg/11.10.1185

[3] Dandona L, Williams JD, Williams BC, Rao GN (1998) Population-based assessment of childhood blindness in southern India. Arch Ophthalmol 116: 545–546. 9565065

[4] Tanwar M, Dada T, Sihota R, Dada R (2009) Identification of four novel cytochrome P4501B1 mutations (p.I94X, p.H279D, p.Q340H, and p.K433K) in primary congenital glaucoma patients. Mol Vis 15: 2926–2937. 20057908 PMC2802296

[5] Kim HJ, Suh W, Park SC, Kim CY, Park KH, Kook MS, et al (2011) Mutation spectrum of CYP1B1 and MYOC genes in Korean patients with primary congenital glaucoma. Mol Vis 17: 2093–2101. 21850185 PMC3156779

[6] Bejjani BA, Lewis RA, Tomey KF, Anderson KL, Dueker DK, Jabak M, et al (1998) Mutations in CYP1B1, the gene for cytochrome P4501B1, are the predominant cause of primary congenital glaucoma in Saudi Arabia. Am J Hum Genet 62: 325–333. 9463332 10.1086/301725 PMC1376900

[7] Kakiuchi-Matsumoto T, Isashiki Y, Ohba N, Kimura K, Sonoda S, Unoki K (2001) Cytochrome P450 1B1 gene mutations in Japanese patients with primary congenital glaucoma(1). Am J Ophthalmol 131: 345–350. 11239867 10.1016/s0002-9394(00)00808-4

[8] Khan K, Rudkin A, Parry DA, Burdon KP, McKibbin M, Logan CV, et al (2011) Homozygous mutations in PXDN cause congenital cataract, corneal opacity, and developmental glaucoma. Am J Hum Genet 89: 464–473. 10.1016/j.ajhg.2011.08.005 21907015 PMC3169830

[9] Li H, Durbin R (2009) Fast and accurate short read alignment with Burrows-Wheeler transform. Bioinformatics 25: 1754–1760. 10.1093/bioinformatics/btp324 19451168 PMC2705234

[10] McKenna A, Hanna M, Banks E, Sivachenko A, Cibulskis K, Kernytsky A, et al (2010) The Genome Analysis Toolkit: a MapReduce framework for analyzing next-generation DNA sequencing data. Genome Res 20: 1297–1303. 10.1101/gr.107524.110 20644199 PMC2928508

[11] Jun G, Flickinger M, Hetrick KN, Romm JM, Doheny KF, Abecasis GR, et al (2012) Detecting and estimating contamination of human DNA samples in sequencing and array-based genotype data. Am J Hum Genet 91: 839–848. 10.1016/j.ajhg.2012.09.004 23103226 PMC3487130

[12] Wang K, Li M, Hakonarson H (2010) ANNOVAR: functional annotation of genetic variants from high-throughput sequencing data. Nucleic Acids Res 38: e164 10.1093/nar/gkq603 20601685 PMC2938201

[13] Ali M, McKibbin M, Booth A, Parry DA, Jain P, Riazuddin SA, et al (2009) Null mutations in LTBP2 cause primary congenital glaucoma. Am J Hum Genet 84: 664–671. 10.1016/j.ajhg.2009.03.017 19361779 PMC2680998

[14] Narooie-Nejad M, Paylakhi SH, Shojaee S, Fazlali Z, Rezaei Kanavi M, Nilforushan N, et al (2009) Loss of function mutations in the gene encoding latent transforming growth factor beta binding protein 2, LTBP2, cause primary congenital glaucoma. Hum Mol Genet 18: 3969–3977. 10.1093/hmg/ddp338 19656777

[15] Lim SH, Tran-Viet KN, Yanovitch TL, Freedman SF, Klemm T, Call W, et al (2013) CYP1B1, MYOC, and LTBP2 mutations in primary congenital glaucoma patients in the United States. Am J Ophthalmol 155: 508–517 e505 10.1016/j.ajo.2012.09.012 23218701 PMC3736560

[16] Abu-Amero KK, Osman EA, Mousa A, Wheeler J, Whigham B, Allingham RR, et al (2011) Screening of CYP1B1 and LTBP2 genes in Saudi families with primary congenital glaucoma: genotype-phenotype correlation. Mol Vis 17: 2911–2919. 22128238 PMC3224840

[17] Sharafieh R, Child AH, Khaw PT, Fleck B, Sarfarazi M (2013) LTBP2 gene analysis in the GLC3C-linked family and 94 CYP1B1-negative cases with primary congenital glaucoma. Ophthalmic Genet 34: 14–20. 10.3109/13816810.2012.716486 22924778

[18] Mohanty K, Tanwar M, Dada R, Dada T (2013) Screening of the LTBP2 gene in a north Indian population with primary congenital glaucoma. Mol Vis 19: 78–84. 23378721 PMC3559091

[19] Shipley JM, Mecham RP, Maus E, Bonadio J, Rosenbloom J, McCarthy RT, et al (2000) Developmental expression of latent transforming growth factor beta binding protein 2 and its requirement early in mouse development. Mol Cell Biol 20: 4879–4887. 10848613 10.1128/mcb.20.13.4879-4887.2000 PMC85939

[20] Gibson MA, Hatzinikolas G, Davis EC, Baker E, Sutherland GR, Mecham RP (1995) Bovine latent transforming growth factor beta 1-binding protein 2: molecular cloning, identification of tissue isoforms, and immunolocalization to elastin-associated microfibrils. Mol Cell Biol 15: 6932–6942. 8524260 10.1128/mcb.15.12.6932 PMC230948

[21] Vehvilainen P, Hyytiainen M, Keski-Oja J (2009) Matrix association of latent TGF-beta binding protein-2 (LTBP-2) is dependent on fibrillin-1. J Cell Physiol 221: 586–593. 10.1002/jcp.21888 19681046

[22] Hirani R, Hanssen E, Gibson MA (2007) LTBP-2 specifically interacts with the amino-terminal region of fibrillin-1 and competes with LTBP-1 for binding to this microfibrillar protein. Matrix Biol 26: 213–223. 17293099 10.1016/j.matbio.2006.12.006

[23] Desir J, Sznajer Y, Depasse F, Roulez F, Schrooyen M, Meire F, et al (2010) LTBP2 null mutations in an autosomal recessive ocular syndrome with megalocornea, spherophakia, and secondary glaucoma. Eur J Hum Genet 18: 761–767. 10.1038/ejhg.2010.11 20179738 PMC2987369

[24] Kumar A, Duvvari MR, Prabhakaran VC, Shetty JS, Murthy GJ, Blanton SH (2010) A homozygous mutation in LTBP2 causes isolated microspherophakia. Hum Genet 128: 365–371. 10.1007/s00439-010-0858-8 20617341

[25] Haji-Seyed-Javadi R, Jelodari-Mamaghani S, Paylakhi SH, Yazdani S, Nilforushan N, Fan JB, et al (2012) LTBP2 mutations cause Weill-Marchesani and Weill-Marchesani-like syndrome and affect disruptions in the extracellular matrix. Hum Mutat 33: 1182–1187. 10.1002/humu.22105 22539340

[26] Choi A, Lao R, Ling-Fung Tang P, Wan E, Mayer W, Bardakjian T, et al (2015) Novel mutations in PXDN cause microphthalmia and anterior segment dysgenesis. Eur J Hum Genet 23: 337–341. 10.1038/ejhg.2014.119 24939590 PMC4326713

[27] Yan X, Sabrautzki S, Horsch M, Fuchs H, Gailus-Durner V, Beckers J, et al (2014) Peroxidasin is essential for eye development in the mouse. Hum Mol Genet 23: 5597–5614. 10.1093/hmg/ddu274 24895407 PMC4189897

[28] Lazar E, Peterfi Z, Sirokmany G, Kovacs HA, Klement E, Medzihradszky KF, et al (2015) Structure-function analysis of peroxidasin provides insight into the mechanism of collagen IV crosslinking. Free Radic Biol Med 83: 273–282. 10.1016/j.freeradbiomed.2015.02.015 25708780

[29] Vranka JA, Kelley MJ, Acott TS, Keller KE. (2015) Extracellular matrix in the trabecular meshwork: intraocular pressure regulation and dysregulation in glaucoma. Exp Eye Res 33:112–25. 10.1016/j.exer.2014.07.014 PMC4379427 25819459

